# Curative Effects of* Suhuang Zhike Capsule* on Postinfectious Cough: A Meta-Analysis of Randomized Trials

**DOI:** 10.1155/2016/8325162

**Published:** 2016-08-28

**Authors:** Pinpin Ding, Qian Wang, Jing Yao, Xian-Mei Zhou, Jia Zhu

**Affiliations:** ^1^Nanjing University of Chinese Medicine, Nanjing, Jiangsu 210023, China; ^2^Department of Respiratory Medicine, Jiangsu Province Hospital of Chinese Medicine, Affiliated Hospital of Nanjing University of Chinese Medicine, Nanjing, Jiangsu 210029, China

## Abstract

*Objective*. In this paper, we intended to systematically evaluate the efficacy of* Suhuang Zhike Capsule* (SZC) on postinfectious cough (PIC) in adults (age > 18).* Methods*. MEDLINE (PubMed), Chinese National Knowledge Infrastructure (CNKI), Cqvip Database (VIP), and Wanfang Database were researched for the randomized controlled trials (RCTs) of SZC for PIC. The search was limited to human studies, using the search keywords or free-text terms “cough,” “post-infectious cough,” “postinfectious cough,” “post-cold cough,” “postviral cough,” “postcold cough,” “*Suhuang Zhike capsule,*” “Chinese Medicine,” and “randomized clinical trials”. Two reviewers individually extracted data from the included RCTs and then the extracted data were analyzed using Review Manager 5.3 software.* Results*. Seven RCTs involving 573 patients entered the inclusion criteria. Findings suggested that, compared with western conventional medicine (WCM) and other Chinese medicine, SZC could effectively improve the efficacy rate (OR 2.68, 95%  CI, 1.48–4.84, *P* = 0.001; OR 4.86, 95%  CI, 1.50–15.73, *P* = 0.008, separately). Moreover, SZC could also improve the efficacy rate of Chinese medicine symptom (MD −0.74, 95%  CI, −1.46~−0.02, *P* = 0.04). However, in terms of cough relief time, more evidence is needed to prove that SZC have an earlier antitussive effect (MD −1.31, 95%  CI, −3.06~0.45, *P* = 0.14).* Conclusion*. The current evidence shows that SZC is effective in the treatment of PIC in adults and can significantly improve the effective rate of Chinese medicine symptoms.

## 1. Introduction

Cough is one of the most common complaints for which patients seek medical attention. According to the durations of coughing, cough was classified as acute cough (<3 weeks), subacute cough (3–8 weeks), and chronic cough (>8 weeks) [1]. Postinfectious cough (PIC), or postcold cough, is supposed to be the most common cause of subacute cough [2]. The specific infection causing PIC has not been clearly recognized. Respiratory viruses (particularly respiratory syncytial virus, influenza, parainfluenza, and adenovirus),* M. pneumoniae*,* Chlamydophila pneumoniae* strain TWAR,* Moraxella catarrhalis*, and* B. pertussis* have all been implicated [3–10]. While the pathogenesis of the PIC is not known, it has been thought to be due to the extensive disruption of epithelial integrity, widespread airway inflammation, bronchial hyper responsiveness, and cough hypersensitivity [1, 11–18]. Patients, who suffered from PIC, complain of a persistent cough after experiencing the acute symptoms of an upper respiratory tract infection [19]. Studies showed that, in respiratory outpatients, the frequency of PIC ranged from 11% to 25%, which increased to the range from 25 to 50% during outbreaks of atypical pathogens infections [20–22]. The main symptoms of PIC are irritating nonproductive cough or producing a small amount of white mucus sputum. It may last more than 3 weeks but no more than 8 weeks with no abnormalities on their chest X-ray films. Up to now, antibechic, antitussive drugs, antihistamines, antibiotics, and corticosteroids are being commonly prescribed in western conventional medication (WCM). A survey, conducted in the US, demonstrated that 360 million dollars were spent on over-the-counter (OTC) medications for chronic cough every year, and more than 10 billion dollars were spent on the treatment of cough globally [23]. However, little, if any, satisfactory results were obtained.

Based on the theory of “treatment according to different syndromes,” satisfactory clinical curative effects of traditional Chinese medicine (TCM) have been shown [24–27]. Currently, a systematic review showed that, compared with western medicine, Chinese herbal medicine has obvious advantages in the treatment of PIC [28]. According to the theory of TCM, cough mainly results from dysregulation of dispersing and descending lung Qi and can be divided into two categories: exogenous cough and endogenous cough. PIC, equivalent to the category of exogenous cough, is caused by invasion of external evil factors, such as wind-evil, cold-evil, wet-evil, dryness-evil, summer-damp-evil, and fire-evil. Clinical practice and TCM syndrome research indicate that the “wind-evil invading the lung” syndrome is the most common type [25, 29, 30].

PIC is characterized by paroxysmal nonproductive cough and throat itching to cough and aggravated when suffering from foreign odors. All of these features are in accordance with the characteristics of “wind” in TCM. Professor Chao, who is inspired by the theory of anemogenous cough in historical records of TCM, classified PIC as anemogenous cough. According to Professor Chao's wind-cough theory, the TCM pathogenesis of PIC includes pathogenic wind invading lung, pathogenic wind hindering Lung Meridian, Lung Qi obstruction, the obstruction of tracheobronchial, and the contracture of the tracheobronchial. Therefore, Professor Chao holds the view that therapeutic principles, such as wind-dispelling, lung-diffusing, soothing urgency, and suppressing cough, should be commonly applied for “wind-cough” [31]. Thus,* Suhuang Zhike Capsule* (SZC) was invented, which was mass manufactured by Beijing Haiyan Pharmaceutical Industry Co., Ltd., and was approved to treat cough variant asthma (CVA) and PIC by China Food and Drug Administration (CFDA) in 2009 (number Z20103075). Orally taking SZC at a dose of 1.35 g (3 capsules) per time, 3 times per day, was recommended. The course of treatment was 7 to 14 days. It has been commonly prescribed and has gotten obvious curative effects in China. SZC consists of* Mahuang* (*Ephedra sinica *Stapf.),* Zisu* (*Perilla frutescens* (L.) Britt.),* Dilong* (*Pheretima aspergillum* (E. Perrier)),* Pipaye* (*Eriobotrya japonica *(Thunb.) Lindl.),* Chantui* (*Cryptotympana pustulata *Fabricius),* Qianhu* (*Peucedanum praeruptorum *Dunn),* Niubangzi* (*Arctium lappa *L.), and* Wuweizi* (*Schisandra chinesis *(Turcz.) Baill.). Modern pharmacology experiments manifested that ingredients of SZC,* Zisu*,* Mahuang*, and* Dilong,* for example, had anti-inflammatory activities, antitussive effect, and antiasthmatic effect in animals [32–35], and clinical trials also demonstrated that it can relieve cough, soothe wheezing, work as an expectorant, and regulate immunity [36, 37].

Nowadays, an increasing number of clinical trials on SZC for PIC have been reported. However, most of them are randomized controlled trials (RCTs) with small sample sizes, which make it difficult to get reliable conclusions. Therefore, this current systematic review aims to collect the evidence from RCTs to evaluate the therapeutic effect of SZC in the management of PIC.

## 2. Methods

### 2.1. Research Protocol

All methods were performed according to a predefined protocol, which consisted of the search databases, search strategies, and inclusion criteria. The detailed research question included study design, patient characteristics, interventions, and languages.

### 2.2. Database and Search Strategies

A comprehensive systematic literature search was carried out on the main scientific electronic databases by two reviewers (Pinpin Ding and Qian Wang) independently. The preliminary electronic databases we searched are MEDLINE (PubMed), Chinese National Knowledge Infrastructure (CNKI), Cqvip Database (VIP), and Wanfang Database. Keywords or free-text terms we utilized are the following: “post-infectious cough,” “postinfectious cough,” “post-cold cough,” “postviral cough,” “postcold cough,” “*Suhuang Zhike capsule,*” “Chinese Medicine,” and “randomized clinical trials”. Two reviewers independently identified studies.

### 2.3. Inclusion Criteria

Studies included in this meta-analysis had to meet all of the following criteria: (a) Types of studies: only clinical RCTs that were published from their inception to November 1, 2015, were eligible, regardless of blinding. (b) Participants: patients diagnosed with PIC, of either gender and any age more than 18, were included. (c) Interventions: only SZC was utilized in experimental group. And in the control group, patients received self-modified herbal formula, other Chinese patent medicine, or placebo as controls were included.

### 2.4. Exclusion Criteria

Researches were ruled out if they had one of the following circumstances: (a) patients who had a fever, pharyngitis, and other diseases; (b) patients whose ages were no more than 18; (c) other medicine prescribed in experiment group in addition to SZC.

### 2.5. Outcomes

The primary outcome measures were as follows: (a) the total effective rate (clinical cure rate + obvious effective rate + showing effective rate), (b) the effective rate of Chinese medicine symptoms (clinical cure rate + obvious effective rate + showing effective rate), (c) cough relief time, and (d) adverse reactions.

According to the Guiding Principle of Clinical Research on New Drugs of TCM, the clinical efficacy of TCM was classified as clinical cure, obvious effect, showing effect, and no effect (Table 1) [38]. Nimodipine method was referenced to evaluate the efficacy of Chinese medicine symptoms [39]. And the efficacy was classified as clinical cure, obvious effect, showing effect, and no effect (Table 2).

### 2.6. Studies Selection and Data Extraction

The detailed method followed the reported one [40]. Two reviewers independently screened the titles and abstracts of searching results meeting predefined inclusion criteria to identify potential relevance that required full texts for further identification. The following information was extracted: authors, year of publication, sample size, age and sex of the participants, details of methodological information, details of the interventions, and outcomes measures and adverse reactions. The most detailed reports were selected if a study was quoted by different literatures. Any disagreements were resolved by consensus or by a third reviewer. All articles included were judged by the third reviewer.

### 2.7. Quality Assessment

Methodological quality of RCTs was assessed by two coauthors independently. The criteria we used were the Jadad score criteria [41]. The following three domains were assessed: method of randomization, blinding, dropouts, and withdrawals. Simply, 2 points were allocated if the method of randomization was described and it was appropriately conducted. 1 point was allocated if the method of randomization was not appropriate. 2 points were allocated if the method of blinding was double-blind and elaborated the blinding method. 1 point was allocated if the method of blinding was not appropriate. 1 point was allocated if the study had a description of withdrawals and dropouts; otherwise, no point was allocated if the study did not have a description of withdrawals and dropouts. Maximum number of points is 5. More than 3 points (including 3 points) was considered to be a high quality study; otherwise, it is a low quality study.

### 2.8. Data Analysis

In this review, the Review Manager 5.3 software was utilized for data analysis. Heterogeneity between similar studies is evaluated by chi-square test and *I* ^2^ statistic. If *P* ≥ 0.05 and *I* ^2^ ≤ 50%, then the possibility of heterogeneity between the studies is low, and a fixed-effects model will be used. If *P* < 0.05 and *I* ^2^ > 50%, there is heterogeneity between the studies, and a random-effects model will be employed. The enumeration data is expressed as odds ratio (OR) with 95% confidence interval (CI). The measurement data is expressed as mean difference (MD) with 95% CI. Statistical significant difference was considered as *P* < 0.05.

## 3. Results

### 3.1. Description of Included Studies

**Figure 1:**
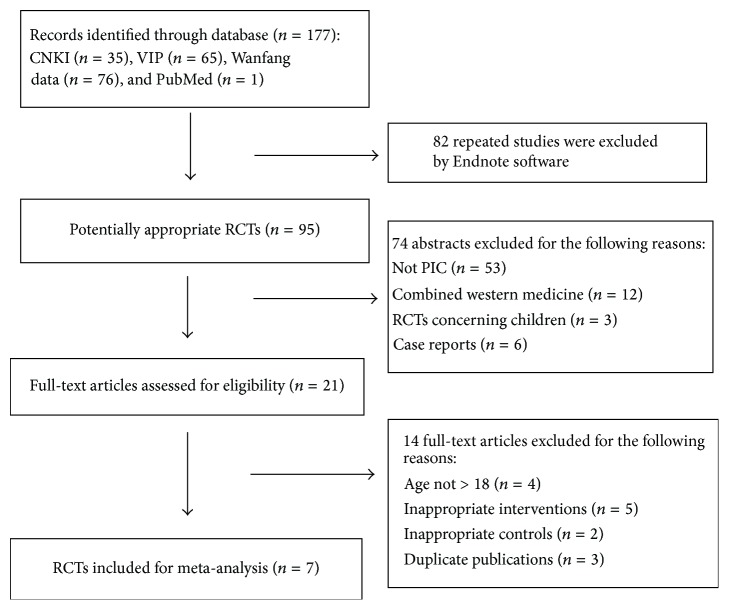
Flow diagram showing the trial selection process for the systematic review. CNKI: Chinese National Knowledge Infrastructure; VIP: Cqvip Database; PubMed: MEDLINE; PIC: postinfectious cough; RCT: randomized controlled trials.

Following the search strategy predefined, 177 potentially relevant citations were screened out: specifically, 35 studies from CNKI, 65 from VIP, 76 from Wanfang Database, and 1 from PubMed. And then 82 duplicated articles were excluded using EndNote X7 software. After reading the titles and abstracts, 74 articles concerning animal experiments, experience reports, and trails carried out on children were eliminated. Then two reviewers carefully read the full text of the rest of the 21 articles; 14 references were excluded as they do not meet all of the inclusion criteria. Thus, a total of 7 eligible trials were accepted for the current meta-analysis [36, 42–47]. A total of 573 participants were involved in the 7 studies, of which 368 patients participated in SZC group and 205 patients in control group (Figure 1 and Table 3).

### 3.2. Methodological Quality of Included RCTs

Based on the inclusion criteria, 7 relevant citations were included in this study. However, there are only 3 studies that used the stochastic indicator method [36, 44, 46]. The rest of them claimed that they have applied a randomized method, but none of them had a specific description. Only one study had precalculated sample size, utilized a double-blind method, elaborated the blinding method, and did an intention-to-treat (ITT) analysis [36]. Of all the seven trials, only 2 mentioned drop-out data [36, 42], but they did not elaborate reasons. The baseline information, such as interventions and outcome measurement, for the treatment and the control group is described in detail. According to the Jadad criteria, only one of them is thought to be a high quality study; the remaining six are low quality studies (Table 4).

### 3.3. Meta-Analysis of SZC Curing the Postinfectious Cough

#### 3.3.1. The Efficacy Rate

**Figure 2:**
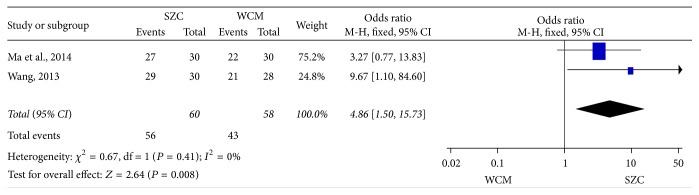
The efficacy rate using SZC versus WCM. SZC:* Suhuang Zhike Capsule*; WCM: western conventional medicine.

*(1) SZC versus WCM*. As is shown in Figure 2, 2 studies talked about the efficacy rate difference between SZC and WCM [42, 45]. 118 cases of patients in total were included (60 patients in the treatment group, 58 patients in the control group). As the 2 studies did not show heterogeneity (chi-square = 0.67, *P* = 0.41, *I* ^2^ = 0%), the fixed-effects model was applied for statistical analysis. Findings suggest that SZC could effectively improve the efficacy rate when compared with WCM (OR 4.86, 95%  CI, 1.50–15.73, *P* = 0.008) (Figure 2).

**Figure 3:**
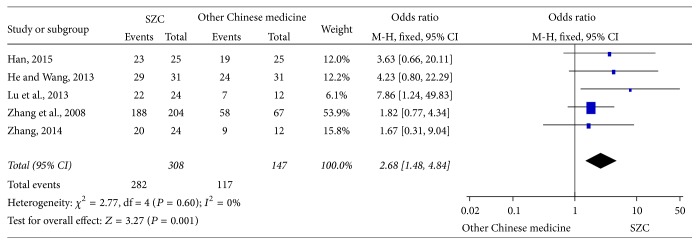
The efficacy rate using SZC versus other Chinese medicine. SZC:* Suhuang Zhike Capsule.*

*(2) SZC versus Other Chinese Medicine*. As is shown in Figure 3, 5 literatures including 455 patients observed the efficiency rate between SZC and other Chinese medicine (308 patients in SZC group, 147 patients in other Chinese medicine group) [36, 43, 44, 46, 47]. After testing for heterogeneity, there is no statistical heterogeneity between these 5 clinical trials (chi-square = 2.77, *P* = 0.60, *I* ^2^ = 0%). Therefore, the fixed-effects model was applied for statistical analysis. Findings suggest that, compared with other Chinese medicine, SZC could effectively improve the efficiency rate (OR 2.68, 95%  CI, 1.48–4.48, *P* = 0.001) (Figure 3).

### 3.4. The Effective Rate of Chinese Medicine Symptoms

**Figure 4:**
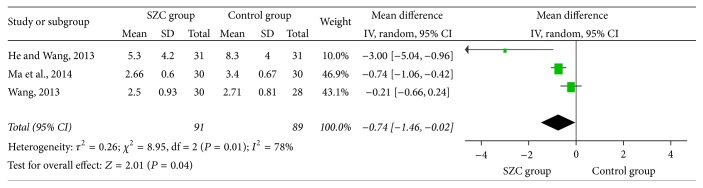
The effective rate of Chinese medicine symptom using SZC versus WCM or other Chinese medicine. SZC:* Suhuang Zhike Capsule*, WCM: western conventional medicine.

There are 3 trials that talked about the effective rate of Chinese medicine symptoms [42, 43, 45]. 180 cases of patients in total were included (91 patients in the treatment group, 89 patients in the control group). As the 3 trials showed heterogeneity (chi-square = 8.95, *P* = 0.01, *I* ^2^ = 78%), the random-effects model was utilized for statistical analysis. Results indicate that there was a significant difference between SZC group and control group (MD −0.74, 95%  CI, −1.46~−0.02, *P* = 0.04) (Figure 4).

### 3.5. Cough Relief Time

**Figure 5:**
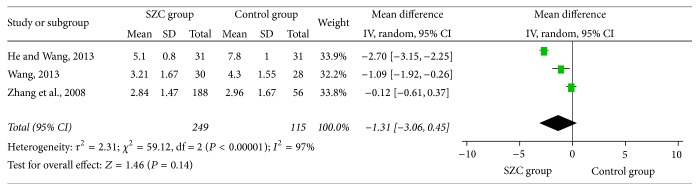
Cough relief time using SZC versus WCM or other Chinese medicine. SZC:* Suhuang Zhike Capsule*; WCM: western conventional medicine.

In all of seven studies, three of them selected cough relief time as one of their outcome measures [36, 42, 43]. 364 cases of patients in total were included (249 patients in the treatment group, 115 patients in the control group). After testing for heterogeneity, there is statistical heterogeneity among these 3 clinical trials (chi-square = 59.12, *P* < 0.00001, *I* ^2^ = 97%). Therefore, the random-effects model was employed in this study. Results imply that there was no significant difference between SZC group and control group (MD −1.31, 95%  CI, −3.06~0.45, *P* = 0.14) (Figure 5).

### 3.6. Adverse Reactions

Only 4 patients experienced nausea and vomiting after taking SZC, and no other adverse reactions were reported [36, 46]. The adverse reactions of commonly used western medicine or Chinese medicine in the control group included headache, drowsiness, dizziness, dry mouth, and fatigue. However, it is difficult to calculate the total incidence of adverse reactions as part of the literatures did not specify the number of reported adverse events.

## 4. Discussion

In this paper, RCT literatures of the efficacy and safety of SZC and commonly used WCM or Chinese medicine in clinical treatment of PIC have been systematically evaluated and analyzed. Results indicate that SZC has advantages in terms of the efficacy rate and the effective rate of Chinese medicine symptoms, and the difference has statistical significance. Also, only 4 patients experienced nausea and vomiting after taking SZC, and no other adverse reactions were reported [36, 46]. Therefore, it is safe in clinics.

However, we should bear in mind that we have some limitations concerning this study. First and foremost, RCTs included in this study are limited and the sample sizes are small. Therefore, it is difficult to rule out the influence of contingency factors. In addition, the overall methodological quality of included RCTs is not very high. Though all of seven literatures declared that they had utilized random method, only three of them elaborated on the details of stochastic method and procedure [36, 44, 46]. Only one study had precalculated sample size, utilized a double-blind method, elaborated the blinding method, and did an ITT analysis [36]. Also, only two trials had a description of withdrawals and dropouts [36, 42]. As a matter of fact, there is a high possibility of selection bias and measurement bias. Therefore, we could virtually impossibly ensure that the trials were properly conducted. Moreover, all the literatures are not uniform in the diagnostic criteria and therapeutic efficacy standards. This brings inexpedience to data merge and statistics. And only one research has the follow-up reports after treatment [42], so it is unable to assess long-term efficacy and safety of SZC. Therefore, the confirmative conclusions are not allowed. High quality, large-sample, multicenter randomized clinical trials still need to be done in the future.

## Figures and Tables

**Table 1:** Subgroups of clinical efficacy of TCM and their descriptions.

Subgroups	Descriptions
Clinical cure	Cough symptoms completely resolved. Score of all main symptoms became zero.
Obvious effect	Cough significantly reduced or disappeared. Score of all main symptoms dropped two levels.
Showing effect	Significantly reduced cough, score of all main symptoms dropped one level, a primary disease dropped two levels, and another dropped one level.
No effect	No obvious mitigation of cough or even having an aggravating cough.

**Table 2:** Subgroups of clinical efficacy of Chinese medicine symptoms and their descriptions.

Subgroups	Descriptions
Clinical cure	*n* ≥ 95%
Obvious effect	70% ≤ *n* < 95%
Showing effect	30% ≤ *n* < 70%
No effect	*n* < 30%

Efficacy index (*n*) = (points before treatment − points after treatment)/points before treatment × 100%. 0 points: no cough; 3 points: coughing occasionally; 6 points: coughing frequently; 9 points: coughing persistently.

**Table 3:** Characteristics of the eligible studies.

Included studies	NO. T/C	Interventions		Duration (days)	Outcomes
		T	C		
Zhang et al. [36]	204/67	SZC	ZhikeNingsou Capsule	7	Efficacy, cough relief time
Wang [42]	30/28	SZC	Compound Methoxyphenamine Hydrochloride Capsule	7	Efficacy, Cough symptom score, cough relief time
He and Wang [43]	31/31	SZC	YifeiZhike Capsule	14	Efficacy, cough relief time
Lu et al. [44]	24/12	SZC	Ke-Yu Syrup	7	Efficacy
Ma et al. [45]	30/30	SZC	Compound Codeine Phosphate Oral Solution	14	Efficacy, Cough symptom score
Zhang [46]	24/12	SZC	Ke-Yu Syrup	7	Efficacy
Han [47]	25/25	SZC	YifeiZhike Capsule	7	Efficacy

T: treatment group, C: control group, SZC: *Suhuang Zhike Capsule*.

**Table 4:** Methodological quality of included RCTs.

RCTs	Randomized method	Blinding	Dropouts or withdrawals	Jadad scores
Zhang et al. [36]	Stochastic Indicator Method	Double-blind	6	5
Wang [42]	Claimed	Unclear	2	2
He and Wang [43]	Claimed	Unclear	No	1
Lu et al. [44]	Stochastic Indicator Method	Unclear	No	2
Ma et al. [45]	Claimed	Unclear	No	1
Zhang [46]	Stochastic Indicator Method	Unclear	No	2
Han [47]	Claimed	Unclear	No	1
